# Targeting to the non-genomic activity of retinoic acid receptor-gamma by acacetin in hepatocellular carcinoma

**DOI:** 10.1038/s41598-017-00233-5

**Published:** 2017-03-23

**Authors:** Wenjun Zeng, Chunyun Zhang, Hongwei Cheng, Yun-Long Wu, Jie Liu, Zekun Chen, Jian-gang Huang, Russell Erick Ericksen, Liqun Chen, Haiping Zhang, Alice Sze Tsai Wong, Xiao-kun Zhang, Weiping Han, Jin-Zhang Zeng

**Affiliations:** 1 Fujian Provincial Key Laboratory of Innovative Drug Target Research and State Key Laboratory of Cellular Stress Biology, School of Pharmaceutical Sciences, Xiamen University, Xiamen, China; 2 Singapore Bioimaging Consortium, Agency for Science, Technology and Research, Singapore, Singapore; 3 Department of Pathology, The First Hospital of Xiamen, Xiamen, China; 4 School of Biological Sciences, University of Hong Kong, Pokfulam Road, Hong Kong, China

## Abstract

We recently demonstrated that retinoic acid receptor-γ (RARγ) is overexpressed and acts as a tumor promoter in hepatocellular carcinoma (HCC). The oncogenic activity of RARγ is mainly attributed to its physiological interaction with p85α regulatory subunit of PI3K leading to constitutive activation of AKT. Here we report RARγ as a negative regulator of p53 signaling and thus extend the oncogenic potential of RARγ to a new role in controlling the balance between AKT and p53. A natural flavonoid acacetin is then identified to be capable of modulating RARγ-dependent AKT-p53 network. It specifically binds to RARγ and inhibits *all-trans* retinoic acid (*at*RA) stimulation of RARγ transactivation. However, the anticancer action of acacetin is independent on its modulation of RARγ-driven transcriptional activity. Acacetin induces cancer cell apoptosis through antagonizing the non-genomic effect of RARγ on AKT and p53. When bound to RARγ, acacetin prevents RARγ from its activation of AKT followed by recovery of the normal p53 signaling. Given the implication of AKT-p53 dysregulation in most HCC, targeting the non-genomic signaling of RARγ that switches AKT-p53 from a pro-survival to a pro-apoptotic program in cancer cells should be a promising strategy for developing novel anti-HCC drugs.

## Introduction

Hepatocellular carcinoma (HCC) remains the leading cause of cancer-related mortality in China and its incidence is also rising in developed countries that were previously known to have low HCC morbidity^1^. It is urgent to develop novel therapeutic approaches for improving HCC prognosis and identification of new molecular targets and pathways is the key^2^.

Nuclear receptors (NRs) have emerged as the second largest class of drug targets^3, 4^. The liver is rich in NRs that are critical for maintaining hepatic homeostasis and function^5^. It is a major storage organ of vitamin A in the body, and itself is the direct target tissue of retinoid metabolites that act through binding to retinoid nuclear receptors, *i.e.*, retinoic acid receptors (RARs) and retinoid X receptors (RXRs)^6^. Impaired retinoid signaling as a result of deficiencies in retinoid contents and/or reduced retinoid receptor expression is often described in chronic liver diseases and HCC^6^. However, the clinical study of β-*at*RA used in HCC patients was demonstrated to induce more aggressive phenotypes rather inhibit tumor growth^7^. Similarly, clinical trials of β-carotene were shown to increase the risk of lung and stomach cancers^8^. In certain maligancies, retinoid receptors even act as a tomurigenesis promoter though without harboring mutations. For example, RARα was shown to be associated with tomoxifen resistant in breast cancer^9^ and RXRα was essential for the oncogenic activity of promyelocytic leukemia (PML) fused with RARα (PML/RARα) in driving leukemia progression *in vivo* ^10^.

The retinoid receptors act as both tumor suppressor and promoter, which may be explained by their different subcellular locations. As a ligand-dependent transcription factor, retinoid receptors are usually located in nucleus, where they activate transcription of retinoid response genes, known as genomic action. However, considerable studies have demonstrated that retinoid receptors can also be translocated into the cytoplasm, where they can actively participate in cell signaling cascades, the so-called non-genomic action. Given the evidence that the non-genomic signaling of nuclear receptors is often amplified and implicated in the pathological processes of cancer and diseases^11, 12^, targeting to this non-genomic signaling is expected to be a promising strategy to develop novel therapies. Thus, the sulindac analogs K-80003, triptolide and xanthone CF31 were recently shown to exert potent anticancer activity through inactivation of the non-genomic oncogenic signaling of truncated RXRα (tRXRα)^13–15^. In addition, the orphan nuclear receptor Nur77 can regulate a switch from cell survival to cell death when it is exported from the nucleus by apoptotic stimuli^16, 17^.

We previously reported our identification of RARγ as an oncogenic protein in HCC^11^. The oncogenic effect of RARγ is not due to its transcriptional regulation but lies in its non-genomic activation of AKT coupled with activation of NF-κB through direct interaction with p85α subunit of PI3K^11^. RARγ-mediated PI3K/AKT and NF-κB signaling can be further enhanced by *at*RA treatment. In animals, overexpression of RARγ confers liver tumor growth, while in clinical trials, *at*RA treatment worsens rather improves the prognosis of HCC^7^. Additionally, RARγ could promote HCC metastasis by down regulating E-cadherin, which has been reported recently^18^. The oncogenic activity of RARγ has also been confirmed in cholangiocarcinoma^19^. Increased RARγ expression is demonstrated to be an indicator of poor prognosis^19^. RARγ has also been shown to activate Wnt/β-catenin and c-Src kinase pathways^19, 20^, suggesting that the propensity of RARγ to regulate tumorigenesis may be through various non-genomic mechanisms. In present study, we wanted to further establish the oncogenic effect of RARγ and identify potential inhibitors of RARγ-mediated oncogenic signaling.

Flavonoids, abundant in many plant species including fruit and vegetables, are excellent leads for drug development^21^. Acacetin (5,7-dihydroxy-4′-methoxyflavone), a flavonoid compound isolated from *Flos Chrysanthemi Indici*, has been shown to have anticancer activity against various malignancies, such as lung cancer, breast cancer and prostate cancer^22–24^, indicating its potential clinical value in cancer treatment^25^. It was reported that acacetin can reduce angiogenesis by blocking HIF-1α/VEGF and PI3K/AKT pathways^26, 27^, and inhibit metastasis by decreasing MMP-2 and u-PA expression^28^. However, the direct intracellular target of acacetin remains to be explored. In this study, we identify RARγ as an essential intracellular target of acacetin. Interestingly, we found that the anticancer effect of acacetin is dependent on modulating RARγ-mediated cross-talk between p53 and AKT, a new non-genomic activity of RARγ.

## Results

### Acacetin specifically targets and binds to RARγ

**Figure 1:**
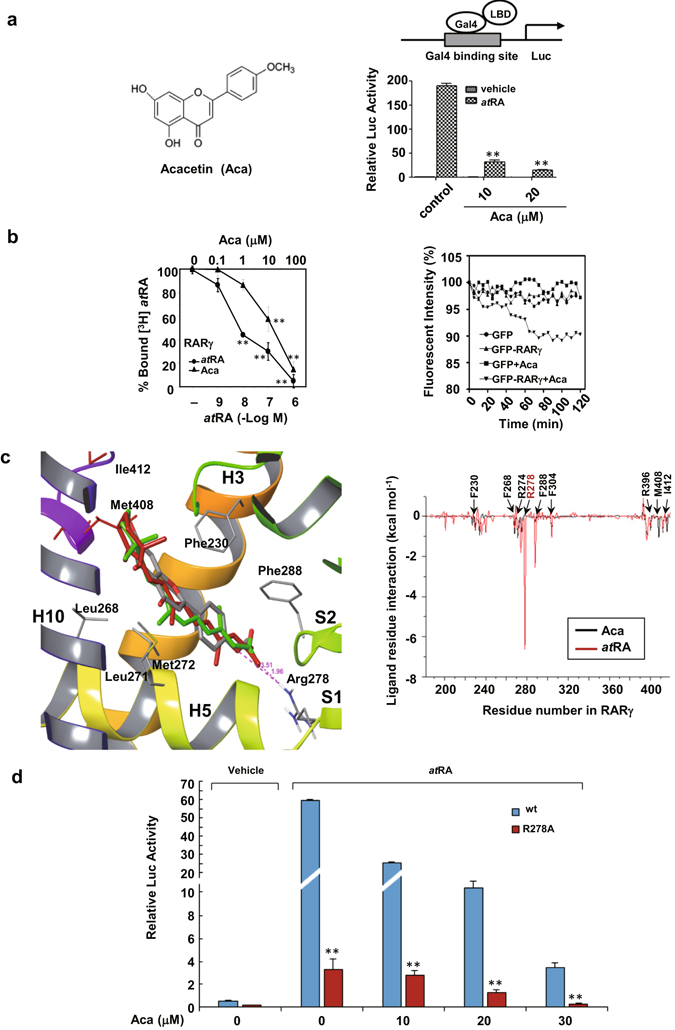
Acacetin antagonizes *at*RA-induced RARγ transcription and binds to RARγ. (**a**) The chemical structure of acacetin is indicated (left). HEK293T cells were co-transfected with pG5-luciferase reporter and pBind-RARγ LBD constructs. The cells were treated with vehicle or acacetin for 12 h in the presence or absence of 10^−7^ M *at*RA and then assayed for luciferase and β-galactosidase activities (right). (**b**) The purified RARγ LBD was incubated with [^3^H]*at*RA in the presence of either *at*RA or acacetin at an increasing concentrations. The capabilities of unlabeled *at*RA and acacetin to displace the radio-labeled [^3^H]*at*RA were evaluated by liquid scintillation counting after 12 h treatment (left). For cell-based assays, HepG2 cells transfected with GFP or GFP-RARγ were exposed to 10 μM acacetin or vehicle for 2 h. The images of live cells incubated at 37 °C were taken every 5 min for assaying the fluorescence intensity (right). (**c**) Structural comparison of acacetin (gray), 9-*cis*-RA (green), and *at*RA (red) on their interactions with the RARγLBD (left). MM-PB/SA was used to calculate the binding free energies in each residue of amino acids 180–450 in the RARγLBD that was docked with *at*RA and acacetin (right). (**d**) HEK293T cells were transfected with Gal4-RARγ/wt or Gal4-RARγ/R278A plasmids and treated with *at*RA (10^−7^ M) in the presence or absence of acacetin at different concentrations. For (**a**), (**b**) and (**d**), the data represent mean ± S.D. of relative luciferase activity generated in triplicate transfections in at least three independent experiments. *p < 0.05 and **p < 0.01 in (**a**) (acacetin + *at*RA *vs at*RA), in (**b**) (*vs* vehicle control) and in (**d**) (R278 *vs* wt).

Acacetin was isolated from traditional Chinese medicinal plant *Flos Chrysanthemi Indici* (Fig. 1a, left panel) and its purity used in this study was ~99%. We firstly identified that acacetin could dose-dependently antagonize *at*RA-inducing luciferase activity in pBind-Gal4-RARγLBD (Fig. 1a, right panel) and RARγ-driven RARE reporter assays, but it had no effect by itself on the basal transcription activity of RARγ (Supplementary Fig. S1a). Except for RARγ, acacetin could not significantly affect *at*RA-stimulated transcription response mediated by RARα or RARβ (Supplementary Fig. S1a). These results suggest that acacetin may bind to RARγ. To determine this possibility, an *in vitro* competitive ligand-receptor binding system was employed. Indeed, both *at*RA and acacetin were demonstrated to displace [^3^H]*at*RA from RARγLBD with IC50 of 6.3 nM and 12.8 μM respectively (Fig. 1b, left panel). To determine the target specificity of acacetin, the binding activities of [^3^H]-labeled *at*RA on RARαLBD and RARβLBD were counted in the presence of unlabeled *at*RA or acacetin. The results showed that both [^3^H]*at*RA-bound complexes could be displaced by unlabeled *at*RA but not by acacetin (Supplementary Fig. S1b). Similarly, the unlabeled 9-*cis*-RA but not acacetin was shown to effectively compete with [^3^H]9-*cis*-RA to bind to RXRαLBD (Supplementary Fig. S1c). The RARγ-binding activity of acacetin was further determined in cell-based fluorescent quenching assays. We showed that the fluorescence taken for 2 h was stable in both GFP-RARγ and GFP-vector transfected cells without treatment. In contrast, the fluorescence in cells transfected with GFP-RARγ but not GFP-vector was significantly quenched by acacetin in a time-dependent manner (Fig. 1b, right panel). Taken together, our results demonstrated that acacetin could specifically target and bind to RARγ.

AutoDock further showed that the total RARγ-docking energy was low for acacetin (−8.68 kcal/mol), indicative of a high receptor-ligand affinity (Supplementary Table S1). Besides, the conformational structures of different crystal complexes of RARγ were comparable when liganded with acacetin, *at*RA and 9-*cis*-RA; all of these compounds fitted well with the same ligand binding pocket of RARγ (Fig. 1c, left panel). MD dynamics simulation demonstrated that acacetin and *at*RA shared several common binding sites including Phe230, Leu268, Leu271, Met272 and Phe288 (Fig. 1c, right panel) by analyzing the docking energy of ligand-per-residue interaction (Supplementary Table S1). Supporting evidence was that virtual point mutations of these residues to Arg resulted in the loss of docking ability.

Classically, *at*RA can form a strong ionic bond with RARγ Arg278 through its carboxylate tail, which is absent in acacetin. To determine the role of Arg278, we mutated it to alanine (R278A). Such mutation greatly impaired *at*RA in activating RARγ-driven reporter expression (114-fold activation for RARγ/wt *vs* 22-fold for Gal4-RARγ/R278A), but did not interfere with the effect of acacetin (Fig. 1d). Instead of interacting with Arg278, acacetin is suggested to bind to two additional residues, Met408 (−1.075 kcal/mol) and Ile412 (−0.796 kcal/mol), which may contribute to stabilization of acacetin/RARγ complex. These findings suggest that acacetin bind to RARγ in a manner different from those classical retinoids.

### RARγ determines the apoptotic effect of acacetin

**Figure 2:**
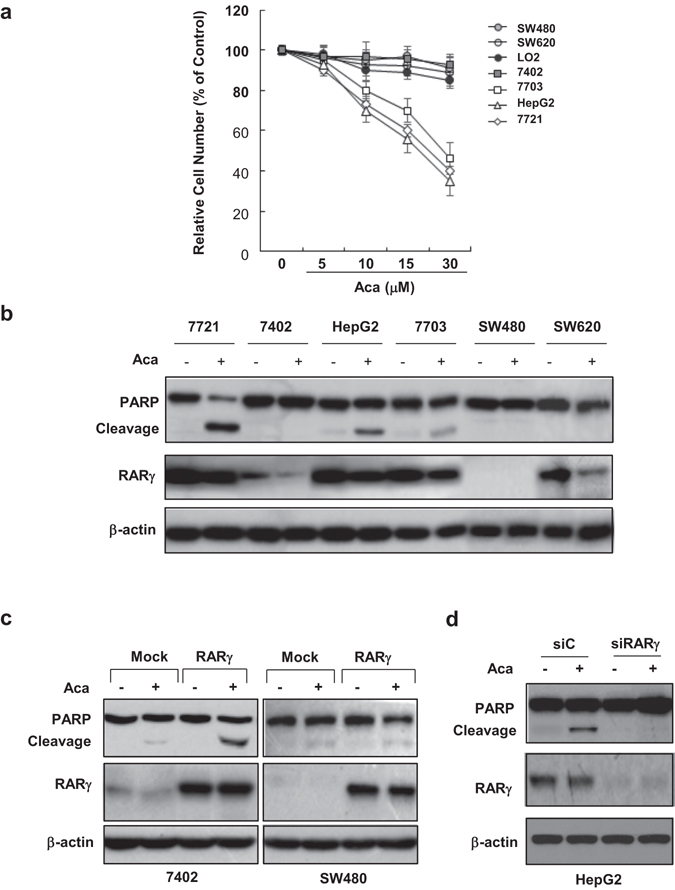
RARγ mediates the anticancer effect of acacetin. (**a**) A number of cancer cell lines were treated with increasing concentrations of acacetin for 48 h and then subjected to MTT assays. The normal liver cell line LO2 was served as control. (**b**) The cancer cells were treated with 15 μM acacetin for 24 h. The cell lysates were blotted for assaying the expression of RARγ and PARP cleavage. β-actin was served as a loading control. (**c**) and (**d**) Bel-7402 and SW480 cells were transfected with myc-RARγ or empty vector (Mock) (**c**), while HepG2 cells were transfected with RARγ siRNA (siRARγ) or control siRNA (siC) (**d**). Transfected cells were treated with 15 μM acacetin or vehicle for 24 h. The expression of RARγ and its association with PARP cleavage induced by acacetin were analyzed by Western blotting. β-actin was served as loading control. All blots were cropped to remove irrelevant or empty lanes.

Acacetin was showed to strongly inhibit the growth of several liver cancer cell lines including HepG2, QGY-7703 and SMMC7721, while Bel-7402 liver cancer cells and normal LO2 liver cells were resistant to acacetin treatment. It was also ineffective in SW480 and SW620 colon cancer cells (Fig. 2a). Western blotting showed that acacetin could strongly induce PARP cleavage in HepG2, QGY-7703 and SMMC7721, but not in Bel-7402, SW480 and SW620, indicating that the anti-cancer effect of acacetin was primarily due to its induction of apoptosis (Fig. 2b). We noted that the cells sensitive to acacetin expressed high levels of RARγ, while those resistant to acacetin treatment expressed low or undetectable RARγ, suggesting that the intracellular levels of RARγ determine the apoptosis-inducing effects of acacetin. An exception was that although SW620 expressed significant amounts of RARγ, the apoptotic effect of acacetin was not induced in this cell line.

RARγ was then transfected into Bel-7402 liver cancer cells and SW480 colon cancer cells, both with very low endogenous RARγ. Interestingly, this transfection only rescued the apoptotic sensitivity of acacetin in Bel-7402, but not SW480 (Fig. 2c), suggesting that RARγ is required for the anticancer activity of acacetin, but its effect is possibly determined by downstream effectors of RARγ. In addition, knocking down RARγ in a sensitive cell line HepG2 by specific siRNA sharply impaired the apoptotic effect of acacetin (Fig. 2d), which further support our conclusion that RARγ is critical for mediation of the action of acacetin.

We then used citral to determine whether inhibition of endogenous retinoic acids could interfere the anticancer activity of acacetin. The sensitive HepG2 cells were treated with 10 or 20 μM acacetin in the absence or presence of 10 or 30 μM citral for 24 h, and then subjected to MTT assays. Our result showed that citral alone could not significantly inhibit the growth of HepG2 cells even at 30 μM. It also could not considerably impact on acacetin-induced growth inhibition of HepG2 cells (Supplementary Fig. S2a). Consistently, adding citral or *at*RA did not affect the effect of acacetin on inducing apoptosis (Supplementary Fig. S2b). Therefore, acacetin-induced cancer cell growth inhibition and apoptosis does not depend on the presence of endogenous or exogenous of RA. Thus, the anticancer activity of acacetin may not be associated with its modulation of the transcriptional program of RARγ, though demonstrated transcriptionally as an antagonist for RARγ.

### RARγ as a novel regulator of p53

**Figure 3:**
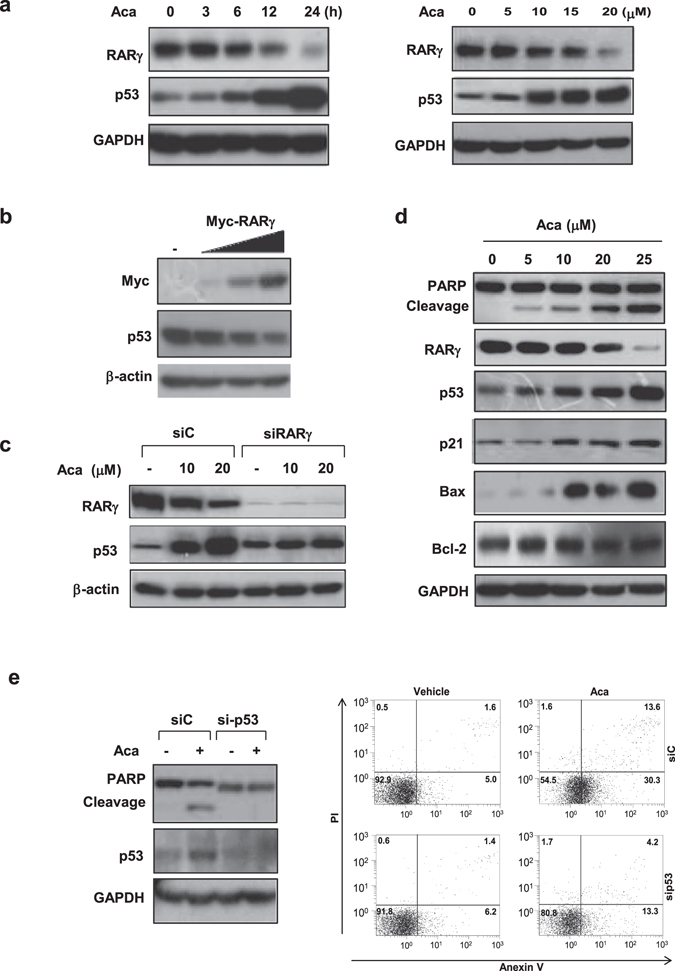
RARγ is a negative regulator of p53. (**a**) HepG2 cells were treated with 15 μM acacetin for different time scales, or with different concentrations of acacetin for 24 h. The effects of acacetin on RARγ and p53 expression were demonstrated. (**b**) HepG2 cells transiently transfected with 10, 30 and 50 ng myc-RARγ were cultured for 32 h. (**c**) HepG2/siRARγ and HepG2/siC cells were treated with 10 μM or 20 μM acacetin for 24 h. The relationship between RARγ and p53 proteins was analyzed. (**d**) HepG2 cells were treated with different concentrations of acacetin for 24 h. The effects of acacetin on PARP cleavage and the expression of RARγ, p53, p21, Bax and Bcl-2 were determined. (**e**) HepG2 cells stably transfected with p53 siRNA (sip53) or scramble siRNA (siC) were treated with 15 μM acacetin for 24 h. The role of p53 in the apoptotic effect of acacetin was determined by PARP cleavage (left) and Annexin V/PI staining (flow cytometry assays, right). The expression levels of all above proteins were essayed by Western blotting. β-actin or GAPDH was served as loading controls. All blots were cropped to remove irrelevant or empty lanes.

Although RARγ was expressed in SW480 (transfected) and SW620 (endogenous), these cell lines were resistant to acacetin treatment. A possible cause may be due to p53 mutations seen in both cell lines. We thus determined the role of p53 in acacetin’s action. Indeed, treatment of HepG2 cells with acacetin resulted in extensively increased p53 expression in time- and dose-dependent manners, an effect possibly occurring at protein level (Fig. 3a) as it did not affect p53 mRNA level (Supplementary Fig. S3a). Interestingly, p53 elevation by acacetin was closely associated with its reduction of RARγ. We then explored the potential role of RARγ in regulating p53 expression by using HepG2 (with wild-type p53) and SW480 (with mutated p53) cells. In HepG2 cells, overexpression of RARγ could dose-dependently inhibit p53 expression (Fig. 3b), while knocking down of RARγ increased p53 expression and greatly impaired the effect of acacetin on inducing p53 expression (Fig. 3c). In contrast, the high levels of mutated p53 in SW480 appeared resistant to RARγ regulation (Supplementary Fig. S3b). These findings suggest that RARγ is a negative regulator of normal but not mutated p53 signaling. Conversely, p53 could not affect RARγ expression as seen in p53 overexpressed or knocked down cells (Supplementary Fig. S3c and d).

The relationship between RARγ and p53 was also explored in HCC samples. RARγ was highly expressed (RARγ^high^) in 4 of 6 tumor tissues, 3 of which were inversely associated with reduced or undetectable p53 (p53^nor^) (Supplementary Fig. S4a). Strong p53 expression, usually indicative of mutated p53 expression (p53^mut^), was detected in 2 tumor tissues, which did not correlate with RARγ expression (Supplementary Fig. S4b). These findings support the *in vitro* observation that only normal but not mutated p53 is possibly regulated by RARγ.

### p53 is essential for acacetin’s action

p53 upregulation by acacetin was further supported by enhancement of p53 downstream target proteins, Bax and p21, in a dose-dependent manner. In contrast, acacetin did not affect the expression of Bcl-2, an anti-apoptotic protein whose regulation is p53-independent (Fig. 3d). Further, acacetin-induced transcriptions of p21, Bax and MDM2 were abrogated by p53 siRNA (Supplementary Fig. S5). Induction of p53 by acacetin was accompanied by PARP cleavage (Fig. 3d), which effect was blocked by p53 siRNA (Fig. 3e, left panel). Flow cytometry assays further showed that the numbers of apoptotic cells induced by acacetin were sharply reduced from about 43.9% to 17.5% in p53 siRNA-transfected cells based on the sum of both right quadrants (Fig. 3e, right panel). Together, our results demonstrate that p53 is critical for the anticancer activity of acacetin.

### Acacetin inactivates AKT by RARγ

**Figure 4:**
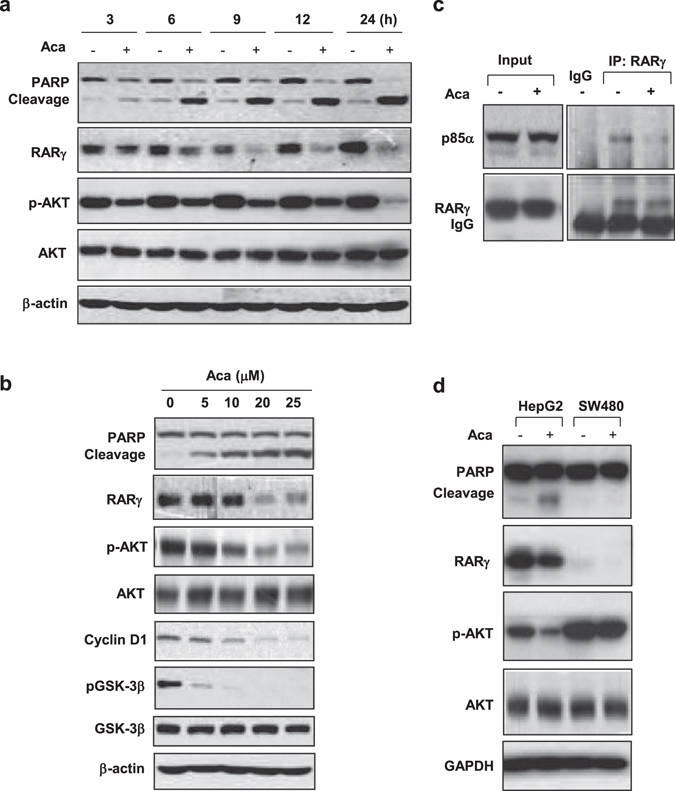
Acacetin induces RARγ-mediated inactivation of AKT. (**a**) and (**b**) HepG2 cells were treated with 15 μM acacetin for different time intervals (**a**), or with different concentrations of acacetin for 24 h (**b**). The levels of total and phosphorylated AKT proteins, together with several downstream proteins were analyzed by immunoblotting. RARγ protein and PARP cleavage were also indicated. (**c**) HepG2 cells were treated with 15 μM acacetin or vehicle for 12 h. The lysates were incubated with anti-RARγ antibody and blotted with anti-RARγ and anti-p85α antibodies. Rabbit IgG was served as Co-IP control. (**d**) HepG2 and SW480 cells were treated with 15 μM acacetin for 24 h. The effects of acacetin on AKT phosphorylation were compared between these two cell lines. All blots were cropped to remove irrelevant or empty lanes.

RARγ-dependent activation of PI3K/AKT pathway was described in several liver cancer cell lines including HepG2 and QGY-7703^11^. We showed here that acacetin could inactivate AKT accompanied by PARP cleavage in time- and dose-dependent manners as indicated in HepG2 (Fig. 4a and b). Consistently, co-immunoprecipitation assays revealed that acacetin could disrupt the constitutive association of RARγ with p85α regulatory subunit of PI3K (Fig. 4c). Further supporting evidence was that the direct GSK-3β substrate of AKT was dephosphorylated and its downstream Cyclin D1 expression was inhibited by acacetin (Fig. 4b). During acacetin-induced AKT inactivation period between 3 and 24 h, the cells became increasingly apoptotic (Fig. 4a). The suppression of AKT activity by acacetin preceded the onset of apoptosis, suggesting that AKT inhibition play a mechanistic role in acacetin-promoted apoptosis. The effects of acacetin on AKT inhibition and apoptosis were also reproducible in all other liver cancer cell lines that expressed high level of RARγ (data not shown). In contrast, SW480 cells that were resistant to acacetin treatment displayed much higher basal AKT activity, which appeared unrelated to RARγ and could not be modulated by acacetin (Fig. 4d).

### AKT as a potent inhibitor of p53

**Figure 5:**
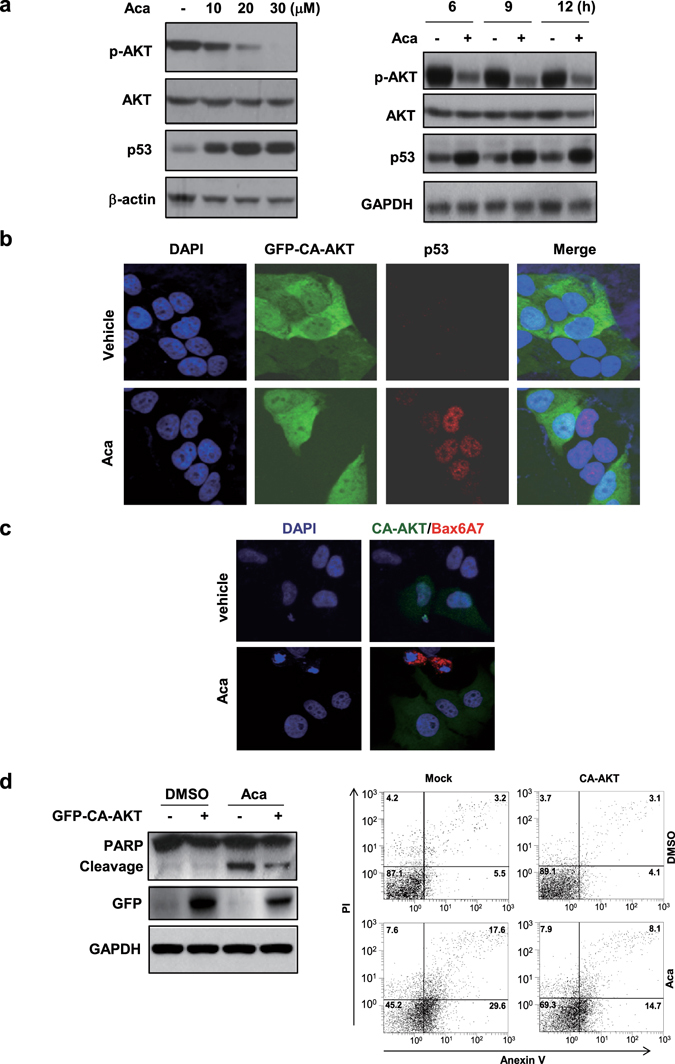
AKT is a potent inhibitor of p53. (**a**) HepG2 cells were treated with different concentrations of acacetin for 6 h or with 15 μM acacetin for different time intervals. The inverse relationship between phosphorylated AKT and p53 was shown in the Western blotting. (**b**) HepG2 cells were transfected with constitutively active AKT (GFP-CA-AKT) and treated with 15 μM acacetin for 12 h. The cells were then immunostained with anti-p53 antibody and co-stained with DAPI. (**c**) HepG2 and its GFP-CA-AKT-transfected cells were treated with 15 μM acacetin for 12 h and then subjected to immuno-staining with conformation-specific Bax/6A7 antibody. The cells were co-stained with DAPI. (**d**) HepG2/CA-AKT and HepG/Mock stable cell lines were treated with 10 μM acacetin for 24 h. The cells were then subjected to immunoblotting (left) or Flow cytometry assays (right). All blots were cropped to remove irrelevant or empty lanes.

As acacetin-induced inactivation of AKT was tightly correlated with its elevation of p53 in both dose and time course studies (Fig. 5a), we sought to determine whether AKT activation can modulate acacetin-induced p53 expression. HepG2 cells were transiently transfected with GFP-CA-AKT, a non-regulatable form of constitutively active AKT. Our results showed that acacetin treatment of non-transfected (GFP^−^) cells induced robust p53 expression, while CA-AKT transfected (GFP^+^) cells failed to show enhanced p53 expression (Fig. 5b). GFP-vector-transfected cells were also used as control, our result showed that GFP-vector had no significant effect on acacetin-induced p53 expression (Supplementary Fig. S7a). These results suggest that acacetin-induced p53 expression is at least partially due to its inactivation of AKT.

### Acacetin induces Bax activation and apoptosis by inactivation of AKT

Acacetin was then shown to extensively induce Bax activation as recognized by a conformation-specific Bax/6A7 antibody (Supplementary Fig. S6). When the cells were transfected GFP-CA-AKT, acacetin-induced Bax/6A7 expression was completely inhibited compared to non-transfected and GFP-vector-transfected cells (Fig. 5c; Supplementary Fig. S7b). Consistently, PARP cleavage by acacetin was significantly inhibited by GFP-CA-AKT (Fig. 5d, left panel). Further, the apoptotic population induced by acacetin was inhibited from about 47.2% to 22.8% in CA-AKT-transfected cells by summing both right quadrants (Fig. 5d, right panel). Our results thus demonstrate that acacetin induce Bax activation and apoptosis through inhibiting AKT activity.

### *In vivo* anti-tumor effect

**Figure 6:**
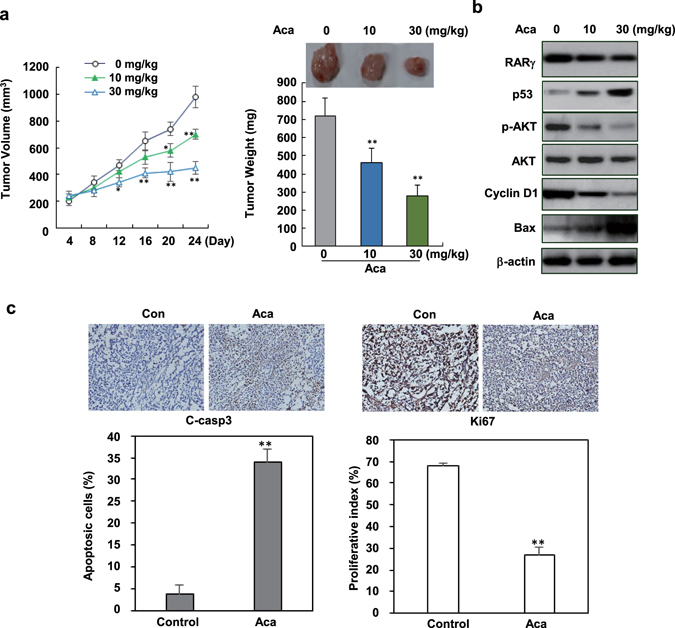
*In vivo* anti-tumor activity of acacetin. (**a**) Mice were subcutaneously transplanted with HepG2/RARγ liver cancer cells. After 1 week, mice with palpable tumors were treated with 10 mg/kg (n = 6) and 30 mg/kg acacetin (n = 6) or vehicle (n = 6) by *i.p.* once every other day. Tumor volume was examined every 4 days after treatment. 3 weeks later of post-treatment, the mice were sacrificed and the tumors were collected for further assays. Representative tumors were shown and the effects of acacetin on tumor weights were evaluated. (**b**) The tumors were lysed and immunoblotted for assaying the expression of RARγ, p53, Bax, Cyclin D1, and the total and phosphorylated AKT proteins. (**c**) The effects of acacetin on the expression of cleaved caspase 3 (indicator of apoptosis) and Ki67 (proliferative index) were conducted by immunostaining. All the slides were co-stained with hematoxylin. The apoptotic and proliferative cell numbers were quantitated. All blots were cropped to remove irrelevant or empty lanes.

HepG2/RARγ, a stable cell line over-expressing transfected RARγ, was characterized and used in animal study (Supplementary Fig. S8). BALB/c mice with HepG2/RARγ xenografts were treated with acacetin or vehicle control respectively. The results showed that acacetin inhibited tumor growth during 3-week treatment resulting in about 36.1% and 61.1% tumor shrink in 10 mg/kg and 30 mg/kg doses respectively compared with vehicle control (Fig. 6a). In acacetin-treated tumors, p53 and Bax were increased, while the phosphorylated AKT and Cyclin D1 proteins were inhibited (Fig. 6b). Consistently, significantly increased cleaved caspase 3 form (apoptosis) and reduced Ki67 expression (proliferative indicator) were extensively induced by acacetin (Fig. 6c).

## Discussion

The deviated localization and enhanced non-genomic signaling of NRs have recently been received great attention. Given its pathological relevance, targeting to the non-genomic signaling pathways of NRs may represent a new direction for drug discovery. Since retinoid receptors are effective drug targets in various malignancies^3, 4^, disclosing their new roles in tumorigenesis will be the key to develop improved anticancer drugs. We recently demonstrated that RARγ is overexpressed and extensively translocated to the cytoplasm in most HCCs, and that its non-genomic activation of AKT contributes to the development of HCC^11^. Our present study demonstrate that a natural flavonoid acacetin can specifically bind RARγ and inhibit its non-genomic, oncogenic activation of AKT. Further, we find a new role for RARγ in determining the survival and death balance though modulating the AKT-p53 network activity.

Firstly, the combination of reporter systems and competitive ligand-receptor binding assays help us to screen and identify acacetin as a new RARγ ligand. Although acacetin can strongly inhibit *at*RA-induced RARγ transcriptional response, the transcriptionally antagonistic role of acacetin does not contribute to its biological outcome as either removing the endogenous RA or adding extra *at*RA did not significantly impact on the apoptotic effect of acacetin in cancer cells. AutoDock and mutagenesis analysis demonstrate that acacetin binds RARγ in a manner distinct of classical retinoids. Unlike *at*RA that binds RARγ highly dependent on its forming a stable salt bridge at Arg278, acacetin employs Met408 and Ile412 to stabilize its interaction with RARγ (Fig. 1c; Supplementary Table S1). Such binding may result in a unique conformational change in RARγ as acacetin treatment strongly prevents RARγ from activation of AKT (Fig. 4c) that are transcriptionally unrelated. Similarly, several new NR ligands like luffariellolide^29^ for RARα, and CF31^15^, *R*-enantiomer etodolac^30^ and K-80003^13^ for RXRα strongly induce cancer cell apoptosis in a non-genomic manner.

Interestingly, RARγ is demonstrated as a negative regulator of p53, a new role that extends its established oncogenic potential. Overexpression of RARγ is consistently associated with downregulation of p53 (Fig. 3b and c). Targeting to RARγ by acacetin leads to strong enhancement of p53 expression and recovery of the normal p53 signaling as demonstrated by its several downstream effectors (Figs 3d and 6b). This is very important as about half of human tumors including HCC that have wild-type p53 display impaired p53 signaling^31, 32^. Since mutant p53 is resistant to RARγ regulation, targeting to RARγ may not be able to restore the normal function of mutant p53.

Retinoids are demonstrated to be a potent p53 inducer^33^, however it is completely unknown whether retinoid receptors are involved in this regulation. Unlike certain retinoids that induce p53 through axin and Stra6 at the transcriptional level^33^, acacetin increases p53 expression at protein level possibly via inactivation of RARγ-dependent AKT activity as the effect can be reversed by overexpression of a constitutively active form of AKT (Fig. 5b). Thus, the anticancer effect of acacetin may be primarily dependent on its capability to target RARγ-mediated balance of p53 and AKT.

The pro-apoptotic Bax protein is likely the key downstream effector of both p53 and AKT. Increased Bax expression by acacetin is dependent on functional p53 signaling as silencing p53 impairs acacetin on inducing Bax transcription (Supplementary Fig. S5). However, Bax activation by acacetin appears due to its inhibition of AKT activity because transfection with a constitutively active CA-AKT completely prevents acacetin from inducing Bax activation (Fig. 5c). Consistently, the mitochondrial translocation and activation of Bax are demonstrated to be regulated by AKT^34, 35^. In addition, reinstating the function of p53 strongly inhibits AKT activity leading to optimal activation of Bax, a method effectively used to overcome cisplatin resistance^36^. Thus, the increased expression and activation of Bax by acacetin may be an indicator of its inducing p53 and AKT pathway crosstalk.

The anticancer effect of acacetin and its induced RARγ-dependent AKT-p53 cross-talk were finally explored *in vivo.* We demonstrated that acacetin can strongly inhibit tumor growth and induce tumor shrink in mice (Fig. 6a), which is closely correlated with its increasing p53 expression accompanied by decreased RARγ and reduced AKT activity (Fig. 6b). Our results suggest that targeting RARγ by acacetin may tip the balance of AKT and p53 from a proliferative (high Ki67 index) to an apoptotic effect (increased cleaved caspase 3) (Fig. 6c). AKT-p53 balance plays a critical role for determining cell fate^37, 38^ and is often dysregulated in many types of tumors including HCC^39–41^. Targeting AKT-p53 network through RARγ may provide a new therapeutic method. Since flavonoids represent a large class of naturally occurring active compounds contained in food and medicinal plants^21^ and have been shown to have potential to combat HCC^42^. Our findings suggest that RARγ-based optimized flavonoid leads may help generate improved anti-HCC drugs.

## Materials and Methods

### Reagents

Lipofectamine 2000 and Annexin V/PI were from Invitrogen (Carlsbad, CA); ECL reagents, goat anti-rabbit and anti-mouse secondary antibodies from Thermo (Rockford, IL); protein A/G Plus-Agarose (sc-2003), polyclonal antibodies against RARγ (C-20, sc-551) and Akt1/2/3 (sc-8312), and monoclonal antibodies against Bax (6A7, sc-23959), p85α (B-9, sc-1637), p53 (sc-126), Bcl-2 (sc-509), p-GSK3β (sc-81495), and Cyclin D1 (sc-20044), and FITC-labeled anti-rabbit IgG from Santa Cruz Biotechnology (Santa Cruz, CA); monoclonal antibodies against GAPDH (G8795) and β-actin (A5441), Citral (CS:5392-40-25), 3-(4,5-dimethylthiazol-2-yl)-2,5-diphenyltetrazolium bromide (MTT) and 4,6-Diamidino-2-phenylindole (DAPI) from Sigma; anti-mouse IgG conjugated with Cy3 from Chemicon international; monoclonal antibodies against pAKT (ser473) (cst-4060) and cleaved caspase-3 (asp175) (#9664), and polyclonal antibody against PARP (#9542) from Cell Signaling Technology (CST); monoclonal antibody against GSK-3β (3D10, ab93926) from Abcam; monoclonal antibody against Ki67 (RB-9043) from Fuzhou Maixin Biotech Co. Ltd., China; PVDF membranes from Millipore; proteinase inhibitor cocktail (11-873-580-001) and PhosSTOP (04-906-837-001) from Roche.

### Preparation of acacetin

*Flos Chrysanthemi Indici* samples collected from Southeast Fujian Province in China were extracted and purified with high performance liquid chromatography (HPLC) for acacetin and analogues^43^. Molecular structures were identified by electron ionization mass spectrometry, Fourier transform IR spectroscopy, and nuclear magnetic resonance analysis.

### Cell lines

Normal human liver cells line LO2, and liver cancer cell lines, QGY-7703, Bel-7402 and SMMC-7721 were from cell bank at Shanghai Cell Biology Institute of Chinese Academy of Sciences, while HepG2 liver cancer cells, SW480 and SW620 colon cancer cells, and HEK293T cells were from American Type Culture Collection (ATCC). Stable cell line HepG2/RARγ with RARγ overexpression was established by transfecting neo-RARγ and selected by 0.5 mg/ml G418.

### Cell culture and transfection

The cells were grown in Dulbecco’s Modified Eagle’s Medium (DMEM) containing 10% fetal bovine serum (FBS), penicillin (100 U/ml) and streptomycin (100 μg/ml). Subconfluent cells with exponential growth were used throughout the experiments. Transfections were carried out by using Lipofectamine 2000 according to the manufacturer’s instructions.

### Reporter assays

HEK293T cells were seeded at the density of 3 × 10^4^ cells per well in 48-well plates and co-transfected with pGL5 luciferase reporter vector (50 ng/well) and pGAL4-RARγLBD expression vector (30 ng/well), or with pGl3-promoter-RARE luciferase reporter vector (40 ng/well), Myc-RARγ (20 ng/well) and pRluc-pcDNA expression vector (10 ng/well). The cells were then treated with vehicle or 10^−7^ M *at*RA in the presence or absence of acacetin at different concentrations for 12 h. Dual-Luciferase Assay System Kit (Promega) was utilized to measure β-galactosidase and luciferase activities.

### Competitive ligand-receptor binding assays

Ligand binding domains (LBDs) derived from RARα, RARβ and RARγ were incubated with [^3^H]*at*RA at 4 °C in the presence or absence of unlabeled *at*RA or acacetin for 12 h. Similarly, RXRα LBD was incubated with [^3^H]9-*cis*-RA combined with unlabeled 9-*cis*-RA or acacetin. Bound [^3^H]*at*RA and [^3^H]9*cis*-RA were quantified in a scintillation counter^15^.

### Fluorescence quenching

Cells were monitored at 37 °C by Leica TCS SP5 laser scanning confocal microscope (Leica Microsystems, Mannheim, Germany) equipped with a thermostat incubator (Tokai Hit, INUB-WELS-F1 series). The fluorescent intensity in GFP- or GFP-RARγ-transfected cells treated with 10 μM acacetin was assayed.

### Molecular docking analysis

Glide (Grid-based Ligand Docking with Energetic) was employed to study ligand-residue interaction (Schrodinger Software Suite, 2014). The force field parameters were determined with Gaussian 03 package at B3LYP/6-31G* level and the molecular dynamics simulations were run with AMBER (version 11.0) program. The ligand-receptor complexes were constructed using Glide and gradually relaxed by 10000 cycles minimization in amber software. The system was slowly heated from 10 K starting temperature to 300 K ending temperature over a period of 50 ps in NVT ensemble. 2-ns MD simulation in NPT ensemble was performed with a balance condition at 300 K temperature and 1 atm pressure.

### siRNA

RARγ siRNA and nonspecific control siRNA used in this study were described previously^11^, p53 siRNA (L-003329) was from DHARMACON.

### RT–PCR analysis

RT-PCR assays were conducted as indicated^11^. The primers were synthesized by Invitrogen (Shanghai Branch), which included: p53 (forward: 5′-CCC TCC TCA GCA TCT TAT CCG AGT GG-3′; reverse: 5′-CTC AGG CGG CTC ATA GGG CAC CAC C-3′), p21 (forward: 5′-ACT GTG ATG CGC TAA TGG CG-3′; reverse: 5′-CCG TGG GAA GGT AGA GCT TG-3′), Bax (forward: 5′-AGA GGT CTT TTT CCG AGT GGC AGC-3′; reverse: 5′-TTC TGA TCA GTT CCG GCA CCT TG-3′), MDM2 (forward: 5′-ATC GGA CTC AGG TAC ATC TG-3′, reverse: 5′-TAC ACC AGC ATC AAG ATC CG-3′), and β-actin (forward: 5′-CAC CAA CTG GGA CGA CAT G-3′; reverse: 5′-GCA CAG CCT GGA TAG CAA C-3′).

### Co-immunoprecipitation (Co-IP) assays and Western blotting

The cells were lysed in 0.5 mL of Co-IP lysis buffer (1% Tritonx-100, 20 mM Hepes, pH 7.6, 150 mM NaCl, 1 mM EDTA) and incubated with anti-RARγ or anti-normal Rabbit IgG antibodies at 4 °C overnight. Protein A/G plus agarose beads were utilized to enrich the immunocomplexes. The cell lysates were electrophoresed on 8% SDS-PAGE and transferred to PVDF membranes and incubated with primary antibodies and secondary antibodies conjugated with horseradish peroxidase. The immunoreactive bands were detected by ECL system. Images in the figures were cropped and adjusted to brightness and contrast by using Fiji and Adobe Photoshop CS6 software (Adobe Systems).

### Immunofluorescence microscopy

Cells fixed with 4% paraformaldehyde were incubated with primary antibodies against Bax 6A7 (1:100), and p53 (1:250), and detected with secondary antibodies conjugated Cy3 (1:200). The cells were co-stained with DAPI to visualize nuclei. Fluorescence images were taken by a Zeiss LSM 510 laser-scanning confocal microscope.

### Human tissues

Surgical resected HCC specimens were collected from the First Hospital of Xiamen and the study was approved by its Ethics Committee. All patients gave informed consent. The slides were immunostained with anti-RARγ (1:200) and p53 (1:250) and counterstained with hematoxylin.

### Flow cytometry analysis

Cells were stained with Annexin V/PI and analyzed by flow cytometry according to the instructions of the manufacturer (Epics Altra; Beckman Coulter, Fullerton, CA, USA).

### MTT assays

Untreated and acacetin-treated cells seeded in 96-well plates were stained with 0.05 mg/ml 3-(4,5-dimethylthiazol-2-yl)-2,5-diphenyltetrazolium bromide (MTT) and measured at 570 nm with an automated microplate reader (Thermo).

### Animal studies

Nude mice (BALB/c, age of 4–5 weeks) were injected subcutaneously with 100 μl of HepG2/RARγ cells (2 × 10^6^). 1 week later, the mice were treated with acacetin (10 mg/kg or 30 mg/kg) or vehicle, with 6 mice in each group, by *i.p.* injection once every other day for 3 weeks. The body weight and tumor sizes were measured every 4 days. At the end of treatment, mice were sacrificed and anatomized, and the tumors were collected. The study was approved by the Animal Care and Use Committee of Xiamen University.

### Ethics Statement

The human tissue collection procedure was approved by the Medical Ethics Committee of the First Hospital of Xiamen. The investigation was approved by Medical Research Ethics Committee of Xiamen University. All mice were handled in accordance with the “Guide for the Care and Use of Laboratory Animals” and the “Principles for the Utilization and Care of Vertebrate Animals”. The protocols were approved by Animal Care and Use Committee of Xiamen University. All methods were performed in accordance with approved guidelines and regulations.

### Statistical analysis

Data analysis was conducted by using Student Test or an analysis of variance, and is presented as the mean ± S.D.

## Electronic supplementary material

Supplementary Datapdf
