# Supplementary material for: Targeting to the non-genomic activity of retinoic acid receptor-gamma by acacetin in hepatocellular carcinoma
Source: Sci Rep. 2017 Mar 23;7:348. doi: 10.1038/s41598-017-00233-5 (PMC5428017; doi:10.1038/s41598-017-00233-5)
Supplement: Supplementary file 1 — Supplementary Datapdf [file 41598_2017_233_MOESM1_ESM.pdf]

SUPPLEMENTARY INFORMATION

Targeting to the non-genomic activity of retinoic acid receptor-gamma by acacetin in hepatocellular carcinoma

Wenjun Zeng[1,#], Chunyun Zhang[1,#], Hongwei Cheng[1,#], Yun-Long Wu[1], Jie Liu[1], Zekun Chen[1], Jian-gang Huang[1], Russell Erick Ericksen[2], Liqun Chen[1], Haiping Zhang[3], Alice Sze Tsai Wong[4,*], Xiao-kun Zhang[1], Weiping Han[2] and Jin-Zhang Zeng[1,*]

[1] Fujian Provincial Key Laboratory of Innovative Drug Target Research and State Key Laboratory of Cellular Stress Biology, School of Pharmaceutical Sciences, Xiamen University, Xiamen, China
[2] Singapore Bioimaging Consortium, Agency for Science, Technology and Research, Singapore.
[3] Department of Pathology, The First Hospital of Xiamen, Xiamen China
[4] School of Biological Sciences, University of Hong Kong, Pokfulam Road, Hong Kong

Author contributions: J.Z.Z. and A.S.W. conceptualized, designed and directed the experiments, obtained funding and drafted the manuscript; W.Z., C.Z., Z.C., J.G.H., H.C., L.C., Y.L.W., J.L., R.E.E. and H.Z. conducted the experiments and data analysis; X.K.Z. and W.H. participated in the research discussion, provided technical support and read the manuscript.

Competing interests: The authors declare no competing financial interests.

[*] Correspondence and requests for materials should be addressed to J.Z.Z (e-mail: jzzeng@xmu.edu.cn) or A.S.W (e-mail: awong1@hku.hk)

[#] These authors made equal contribution to this work

Supplementary Table S1

entry	acacetin		ATRA	
	residue	energy	residue	energy
1	Leu271	-1.075	Arg278	-6.639
2	Met408	-1.059	Phe288	-2.548
3	Ala234	-0.936	Arg274	-1.825
4	Ala397	-0.932	Arg396	-1.209
5	Ile275	-0.895	Ile275	-1.152
6	Leu268	-0.864	Phe304	-1.062
7	Phe304	-0.82	Ala397	-0.973
8	Arg396	-0.805	Leu271	-0.933
9	Ile412	-0.796	Lys236	-0.916
10	Leu416	-0.764	Lys240	-0.813
11	Leu400	-0.708	Ala234	-0.774
12	Phe230	-0.595	Phe230	-0.72
13	Trp227	-0.444	Phe201	-0.705
14	Met272	-0.411	Ile412	-0.693
15	Phe288	-0.287	Leu268	-0.572
16	Leu233	-0.273	Leu400	-0.519
17	Ala394	-0.187	Ser289	-0.458
18	Glu232	-0.164	Lys209	-0.402
19	Met299	-0.146	Lys229	-0.392
20	Pro409	-0.146	Cys237	-0.382

Supplementary Table S1. The energy for the top 20 residues of RARγ in the binding of acacetin and *at*RA. Ligand-residue interaction energy decomposition analysis was performed using the MMPBSA modules of AMBETR 11.1 software. The smaller the value of binding energy, the greater the energy contribution toward the ligand binding. Unit of energy: kcal mol^{-1}.

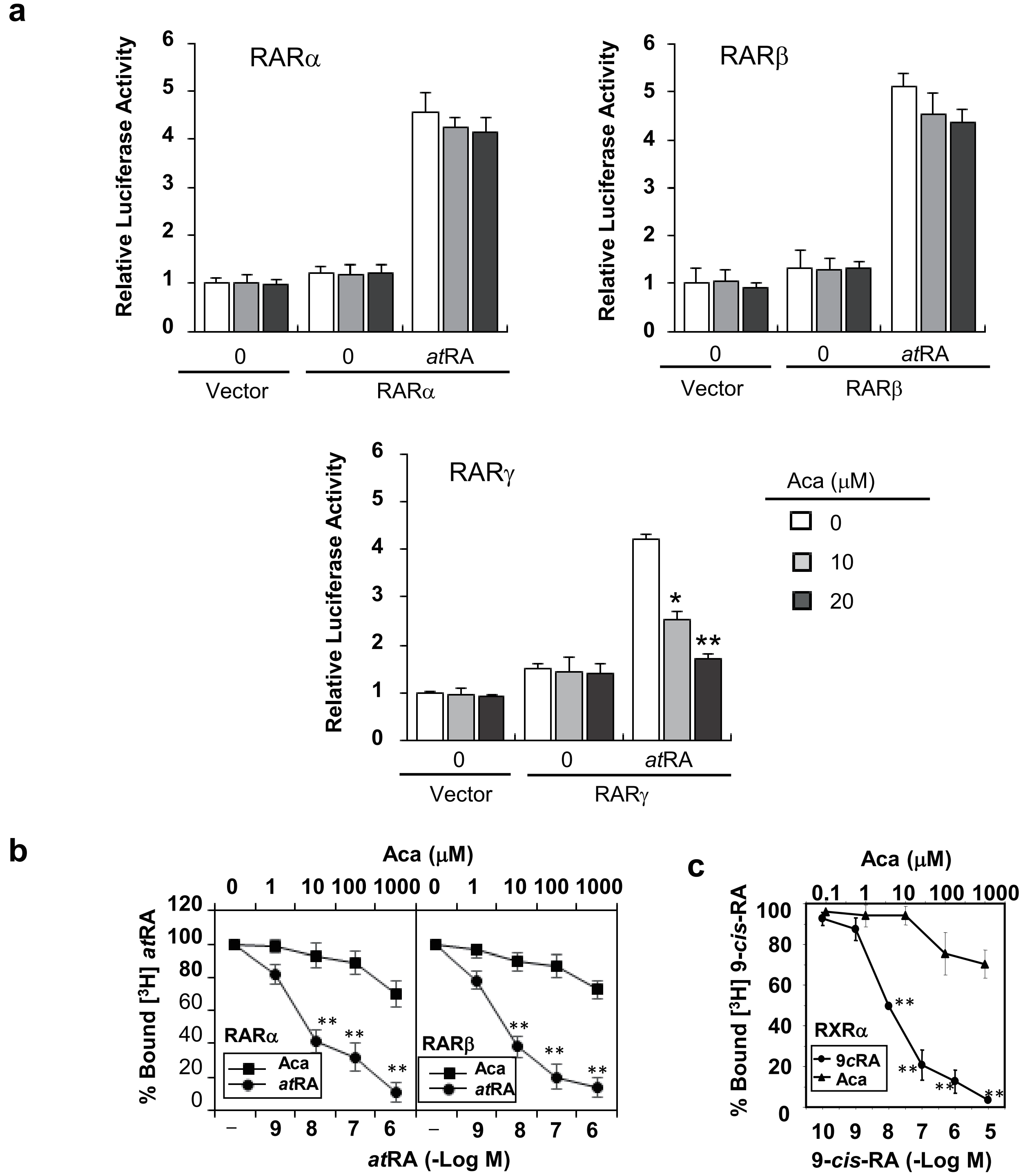

Figure S1. (a) HEK293T cells were co-transfected with RARE reporter combined with full-length of RARα, RARβ or RARγ. The cells were treated with vehicle or acacetin for 12 h and then assayed for luciferase and β-galactosidase activities. **(b)**The purified LBDs of RARα and RARβ were incubated with [^{3}H]*at*RA. The capabilities of unlabeled *at*RA and acacetin to displace the radio-labeled [^{3}H]*at*RA were evaluated by liquid scintillation counting after 12 h treatment. **(c)** Similarly, the ability of acacetin to compete [^{3}H]9-*cis*-RA to bind to RXRα was compared to that of unlabeled 9-*cis*-RA.

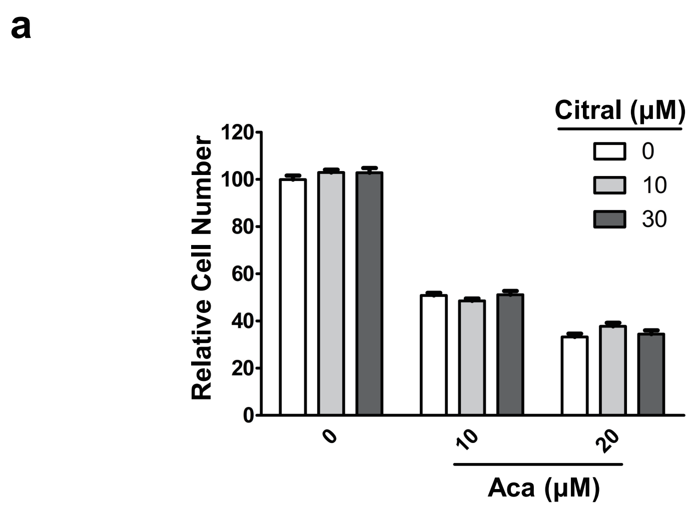

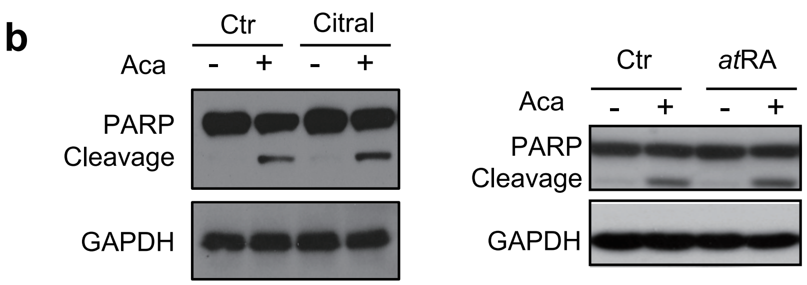

Figure S2. (a) HepG2 cells were treated with 10 or 20 μM acacetin in the absence or presence of 10 or 30 μM citral for 24 h. The cells were then subjected to MTT assays. **(b)** HepG2 cells were treated with 10 μM acacetin in the absence or presence of 0.1 μM *at*RA for 24 h. The cells were then subjected to Western blotting to detect PARP cleavage. GAPDH was served as loading control. All blots were cropped to remove irrelevant or empty lanes.

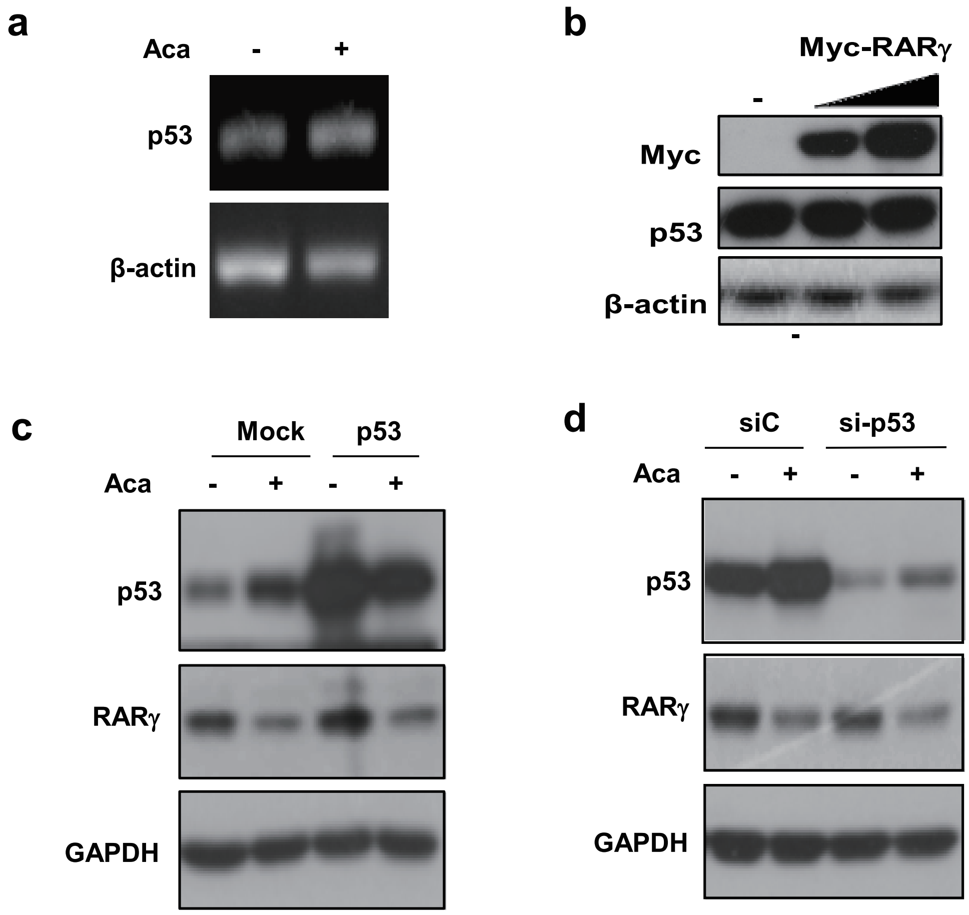

Figure S3. **(a)** HepG2 cells were treated with 15 μM acacetin for 24 h. p53 and β-actin mRNA expressions were assayed. **(b)** SW480 cells were transfected with 40 and 80 ng myc-RARγ. The cells were cultured for 32 h for allowing proteins' expression. The effects of RARγ transfection on p53 expression were determined by Western blotting. **(c)** and **(d)** HepG2 cells were stably transfected with p53 expression vector or p53 siRNA (si-p53). Mock plasmid or scramble siRNA (siC) was served as respective controls. The cells were treated with 15 μM acacetin for 24 h and subujected to Western blotting assays. All blots were cropped to remove irrelevant or empty lanes.

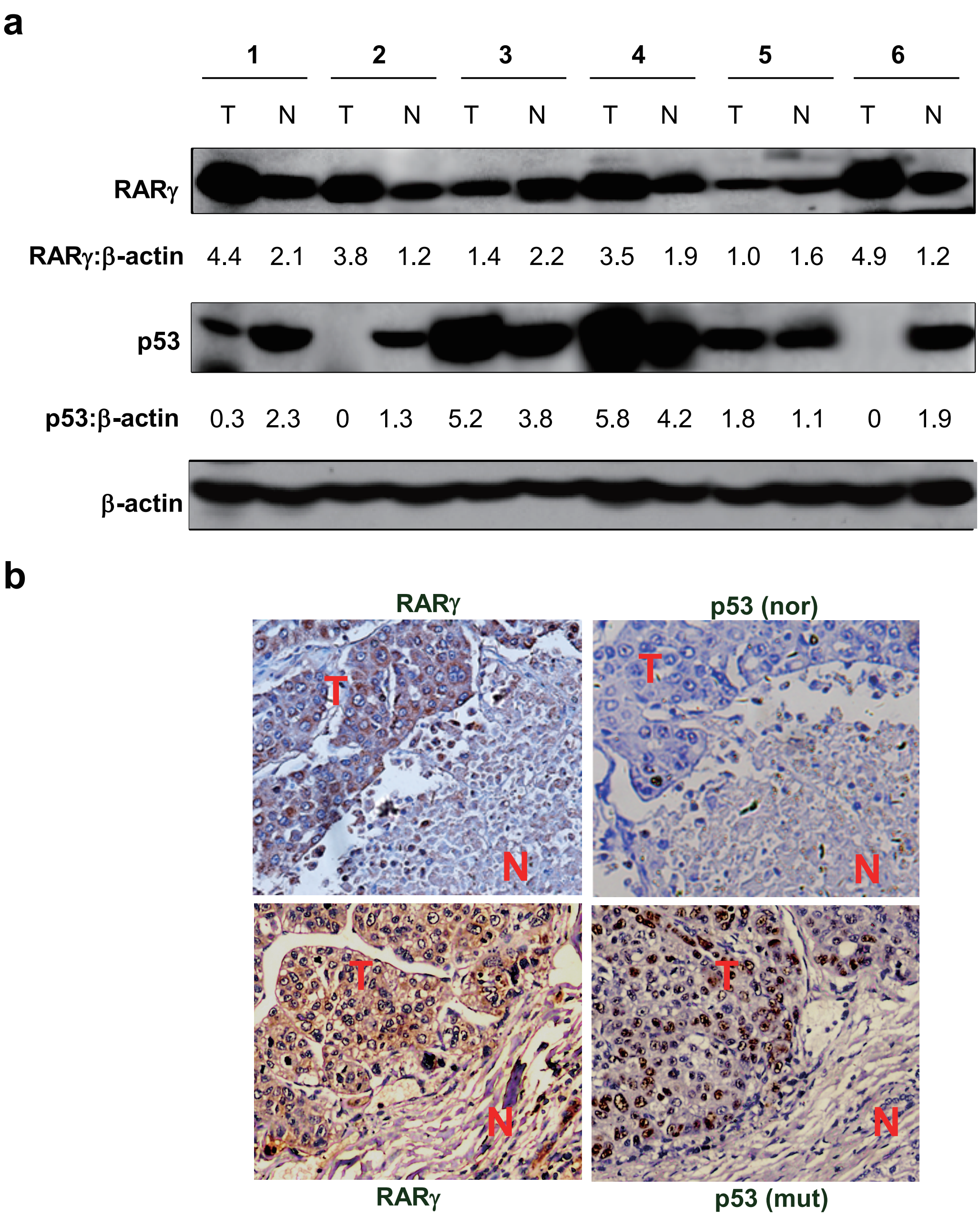

Figure S4. (a) Tumor tissues (T) and their respective non-tumorous tissues (N) were collected from 6 HCC patients. The levels of RARγ and p53 proteins were detected by Western blotting. β-actin or GAPDH was served as loading controls. **(b)** The slides were immunostained with antibodies against RARγ (1:200) and p53 (1:250) and co-stained with hematoxylin. All blots were cropped to remove irrelevant or empty lanes.

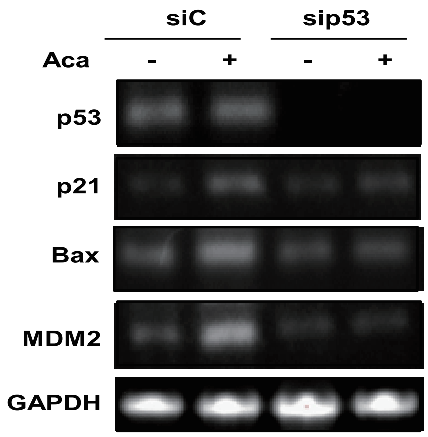

Figure S5. HepG2 cells treated with 15 μM acacetin or vehicle for 12 h and subjected to RT-PCR for assaying the mRNA levels of p53 and its downstream targets including p21, Bax and MDM2. All blots were cropped to remove irrelevant or empty lanes.

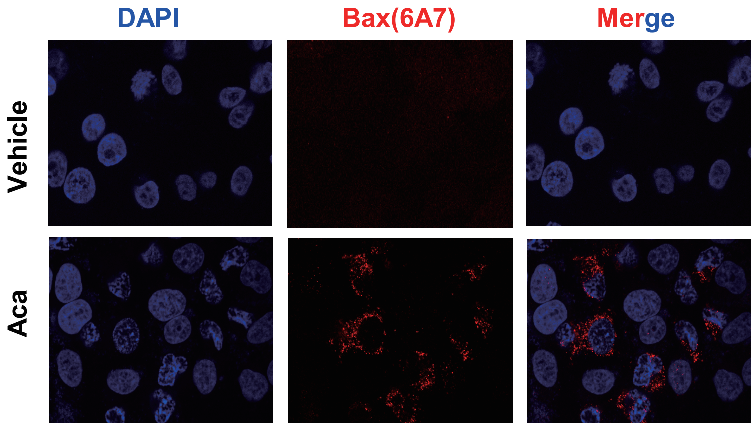

Figure S6. HepG2 cells were treated with 15 μM acacetin for 12 h and then subjected to immuno-staining with conformation-specific Bax/6A7 antibody. The cells were co-stained with DAPI.

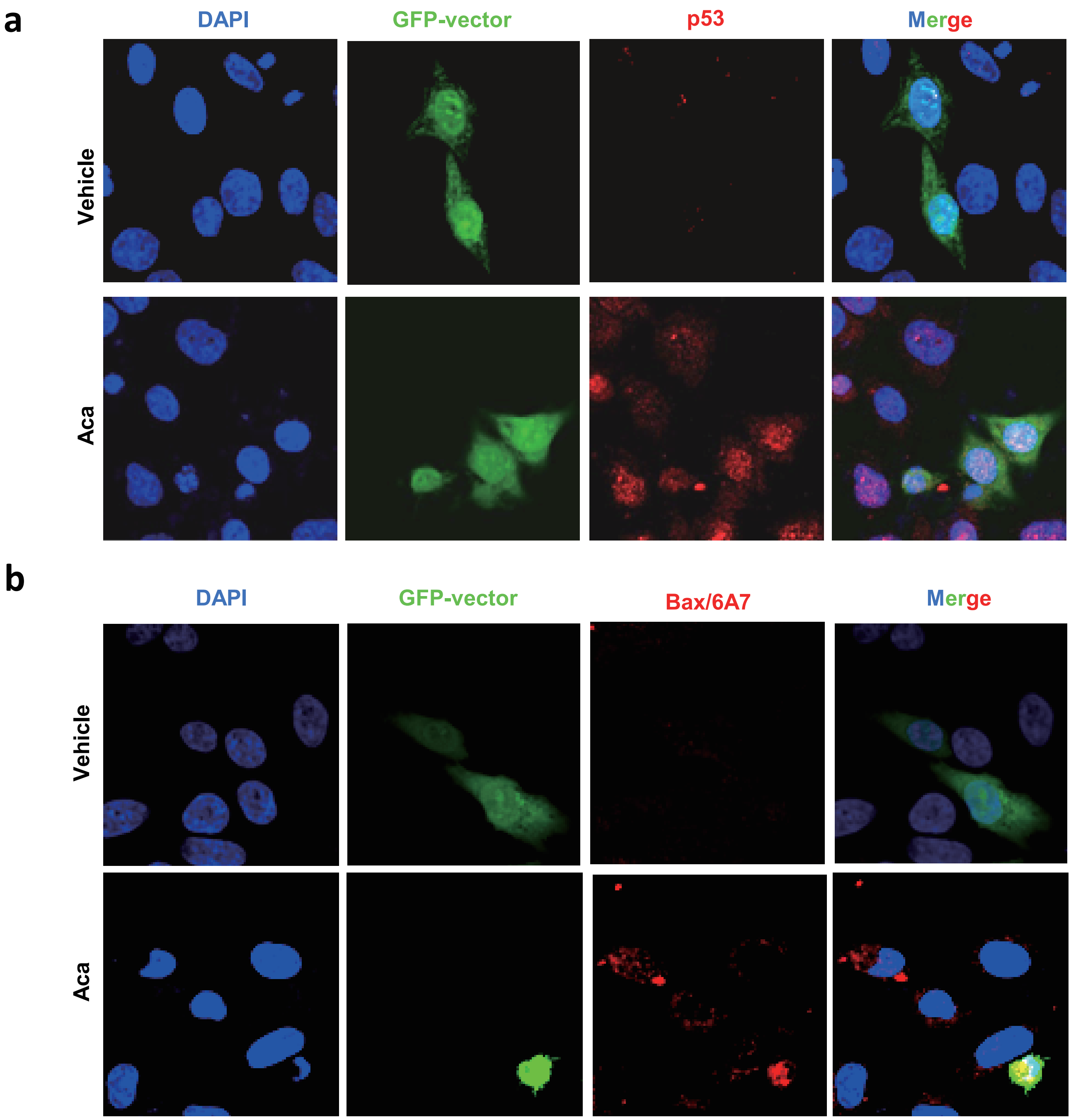

Figure S7. HepG2 cells were transfected equal GFP-vector and treated with vehicle or 15 μM acacetin for 12 h. The cells were then immunostained with anti-p53 **(a)** or conformation-specific Bax/6A7 antibody **(b)**. The cells were co-stained with DAPI to visualize the nucleus. Fluorescence images were taken by laser-scanning confocal microscope.

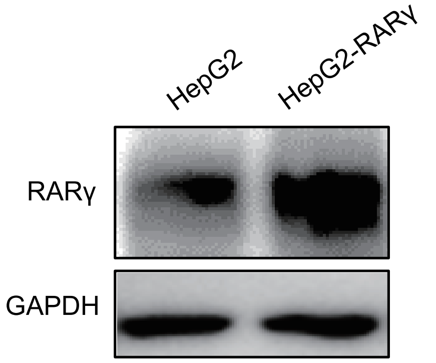

Figure S8. Characterization of HepG2/RARγ stable cell line. The levels of RARγ expression were compared between HepG2/RARγ and HepG2 cells. All blots were cropped to remove irrelevant or empty lanes.
